# Hospital and Institutional News

**Published:** 1912-07-27

**Authors:** 


### HOSPITAL AND INSTITUTIONAL NEWS.

### THE ISSUE AT SHEFFIELD.

An interesting correspondence has been published in the Sheffield papers between the Committee of the Royal Hospital and Sir Henry Burdett. The Committee maintained that Sir Henry was wrong in stating that the hospital had no semi-convalescent home, and Sir Henry, on finding this to be the case, at once tendered an apology. This has now been made a prominent feature in the Sheffield papers, but the mistake itself was one which any thoughtful observer might be excused for making, in view of the over-crowded condition of the children's ward. Such an observer noting this overcrowding would naturally suppose that no semi-convalescent home was in existence, for a properly managed hospital possessing one should never permit it to occur. Sir Henry has asked the Trustees and the Committee for copies of the plans of the hospital, for the cost of which he has offered to pay. The Committee, however, still show no signs of producing these plans. Until they be produced, however, the Committee's case against our report breaks down, since the plans alone can enable an impartial judgment to be delivered upon the points still at issue. The ground plan of the semi-convalescent home appeared in The Hospital of October 17, 1908.

### "THE SCHOOL DOCTOR ON THE GENERAL PRACTITIONER."

Elsewhere in this issue we publish a very carefully reasoned though equally stringent criticism by a school doctor on the general practitioner, whom he argues, for stated reasons, to be unfitted to treat those cases which the school clinic was specially founded to relieve. We have decided to publish his indictment because of its obvious ability and transparent sincerity—qualities which will compel admission, we are sure, even from those most likely to resent the writer's conclusions, or at best, more indulgently to regard his essay in criticism as an imaginary portrait. His main contention appears to be that the "better class" practitioner is not employed now because people suppose that everyone knows something about children. If this be the fact, the writer, perhaps, will be forgiven for emphasising one side of the case, because he calls badly needed attention to that which uncorrected may cripple the better half of the work for which the school clinic has been expressly established. We shall be glad to hear our readers' opinions on the grave issue raised by this article.

### A SANATORIA AUTHORITY FOR LONDON.

A conference of representatives of metropolitan borough councils was presided over by the Mayor of Holborn last week. Professor W. R. Smith moved a resolution to the effect that "the provision and management of sanatoria and hospital accommodation for the treatment of tuberculous persons should also be entrusted to the Metropolitan Asylums Board." He declared that it would be a mistake to hand over the management of sanatoria to the London County Council, and stated that, so far as London was concerned, one bed for 5,000 of the population should be provided, which would mean, roughly, 1,000 beds. The resolution was carried, and it was directed that "tuberculous dispensaries should be established and controlled by metropolitan borough councils, either singly or in groups." The question of a sanatoria authority for London under the Insurance Act was thus definitely discussed, and it will be interesting to see whether the Local Government Board, the Asylums Board, and Mr. Lloyd George agree with the views expressed in the resolutions which were ordered to be sent to them.

### HOSPITAL POLICY IN JERSEY.

Last year (The Hospital, December 9, 1911, page 249) we dealt at some length with the action of the Jersey States in regard to hospital policy in the Channel Islands. We then pointed out the urgent necessity of revitalising public hospital opinion in the island in order to secure an improvement in the hospital conditions to which our attention had been drawn by local correspondents. The matter then centred round the Hôpital Général, which is a poorhouse first and an infirmary secondly. The general discussion which was aroused by the report of the special committee appointed to deal with the hospital question showed that there was active public interest felt in the matter of the hospital, and proved that the profession as well as the lay folk of Jersey realised that hospital accommodation on the island was deficient. Now a further development has occurred. The Jersey General Dispensary and Infirmary has decided, against the advice of the majority of its medical staff, to throw its beds "open to all members of the medical profession practising in the islands, as regards their private patients, in cases where payment is made." This decision was taken on the recommendation of a special committee. The Dean of Jersey, president of the hospital and chairman of the special general meeting at which the matter was decided, did not detail the specific reasons for the recommendation, but hinted that as a result of the donation from the Westaway Fund certain steps had to be taken to broaden the usefulness of the institution, and that that object would be furthered by allowing medical men to send paying patients to be received and treated in private wards. The medical staff pointed out at the meeting that the dispensary cannot honestly be said to be lying empty. With this we are in complete accord. The dispensary has fifteen beds, of which the daily occupied average is eight, which is by no means a bad figure for an institution so peculiarly circumstanced as the Jersey Dispensary. Another point is that the dispensary is designed primarily for poor patients. We have been and are consistently in favour of paying wards attached to voluntary hospitals, but we would not care to see the general wards of a voluntary hospital filled by paying patients to the exclusion of the poor sick, for whose benefit the institution was originally established. When the Jersey Dispensary has built a special paying ward attached to its hospital it can talk of throwing the institution open to the general practitioners of the island; at present such a policy is premature.

### A JERSEY MEDICAL STAFF RESIGNS.

This was fully realised by a member of the committee, who tabled the following sensible amendment: "When further accommodation has been provided, consisting at least of one new ward and two or more private wards with all their suitable accessories, this institution should be then thrown open to all registered medical practitioners for the admission of their private patients on the payment of not less than one guinea per week." This amendment was voted upon, with the result that twelve members were in favour of and twelve against it, and, as the Chairman gave his casting vote against it, the amendment was lost. We think the Chairman's action was a mistake, simply because in such cases it is customary for the Chairman to keep the question open and not to settle it if there is a manifest and almost equally balanced difference of opinion. We now understand that the medical staff of the infirmary has resigned in a body as a protest against the passing of the resolution. This appears to us to be equally a mistake. The proper way is to call another general meeting, to see that it is well attended by those who take a knowledgeable interest in the dispensary, and to discuss the matter calmly. If that be done we do not doubt that the Jersey people will see that it is absurd and foolish to accommodate paying patients in beds which have been provided for the use of the sick poor. In emergency cases there can never be any question as to policy, but to lay down a definite rule, such as the committee has done by passing this resolution, is to overlook entirely the fact that the dispensary is a voluntary charity.

### THE FEEDING OF SCHOOLBOYS.

The speeches at the recent Conference on Diet in Public Schools excited so much interest, and in some instances protest, in the public mind that the National Food Reform Association, of 178 St. Stephen's House, Westminster, will probably have a good sale for the comprehensive and very readable report which they have just issued at 5s. net. Apart from the papers and discussions, which we believe are given in full, specimen dietaries are also printed, and various correspondence, both unpublished matter and excerpts from that which appeared at the time, enlivens these pages with personal opinions. The most practical section is that headed "Display," in which are found specimen menus for meals arranged in weekly rotation, various tuckshop regulations, and certain rather elaborate monographs, notably those of Abbotsholme School, Derbyshire, one of which, the organisation of the sick department, is of more direct interest to our readers. Mr. Charles E. Hecht has taken so much pains as editor to placate the newspapers by his extensive quotations from their innumerable comments that it seems ungracious to draw attention to meticulous errors. One lies in forgetting that the name of this journal consists of two words, the first of which is the definite article. Our vanity finds little to regret in the fact that The Hospital is distinguished from every other journal in the index by being printed in roman type. But the last section of this report has yet to be written. It begins with the "origin and scope" of the Conference. It necessarily omits the effect produced. Let us hope that it will be something more permanently useful to schoolboys than this carefully printed and edited volume. "Words, words, words, we fill our bellies with the east wind: you cannot feed capons so." Schoolboys and Shakespeare are at one on that point. May schoolmasters not forget it when each of them has finished for the last time re-reading this report of his speech! The report is a charming monument to the speeches, but if the question be allowed to drop again it will become their mausoleum and that of the reputations which vented them.

### SECRET REMEDIES FOR SKIN DISEASES.

Some useful evidence has been given before the Select Committee on Secret Remedies by Sir Malcolm Morris, who dealt especially with advertised preparations for skin diseases. He spoke of the ill-effects sometimes produced by such products, and said he had known several instances in which so-called "cures" for ringworm had been used in public elementary schools with the result that an epidemic had been caused. There were remedies advertised for cancer and lupus, the use of which had led to the loss of invaluable time in which other remedies might have been used. He had known a considerable number of such cases during the last thirty-five years. People had come up from the country too late for any treatment and had died perhaps the worst possible form of death that it was possible for a human being to suffer, on account of having used these remedies for long periods in the belief that they would get well ultimately. Sir Malcolm suggested that all proprietary remedies should be submitted to a special Board of Commissioners for analysis, and that the Board should be empowered to act as censors of advertisements, and to forbid the publication of untruthful advertisements. After evidence had been given by Dr. Arthur Whitfield, of King's College Hospital, Mr. H. Sewill, and the Secretary of the White Cross Society, Mr. C. H. Kirby was called. Mr. Kirby has had considerable experience as a solicitor in connection with cases under the Medicine Stamp Duty Acts, the Sale of Food and Drugs Acts, and the Merchandise Marks Act, and he explained the bearing of these Acts on the question into which the Committee is inquiring.

### THE ANTI-HUMANITARIAN AGITATION.

The busybodies who have been pestering the Home Secretary with so-called "medical arguments" against feeding refractory prisoners who refuse food have now been favoured with a copy of the dignified memorandum drawn up by wiser colleagues in the profession who have not allowed sentiment and partisanship to interfere with logic. The signatories of this latter memorial point out that the Home Secretary ought to protect the prison surgeons from the scurrilous and violent denunciations to which they are subjected simply because they do what is manifestly their duty—prevent prisoners from committing suicide. With this suggestion we agree. Mr. McKenna may rest assured that the mass of the profession consists of men and women who possess enough common sense to realise that the feeding of refractory prisoners is not a proceeding directly endangering life or health, and that the hypothetical surmises of sentimentalists must not be allowed to influence prison authorities in condoning breaches of prison discipline. Private practitioners, we venture to think, can do much to eradicate any feeling of horror that the exaggerated accounts of prison "tortures" have caused among the public by explaining to their patients and friends exactly how a nasal or stomach tube is used.

### THE CROYDON HOSPITAL EXTENSION.

The Mayor of Croydon, Alderman Trumble, opened the new wing of the Croydon Hospital. This extension, which is a memorial to King Edward VII., has been built from the designs of Mr. Frank Windsor, at a total cost of £5,500, of which £4,000 has been raised. The new wing comprises a ward with eighteen cots, an isolation department, kitchen, and nurses' bedrooms, while additions have been made also to the Royal Alfred Wing. Sir Frederick Edridge, the chairman of the committee, announced that he had received the promise of £1,000 on condition that the remaining balance were subscribed, and Alderman Trumble stated that £250 of this had been offered that very afternoon. A dedication service was held by the Bishop of Croydon, assisted by Canon White-Thomson.

### THE LISTER MEMORIAL.

On Monday last a meeting was held by the committee formed by the Presidents of the Royal Society and of the Royal College of Surgeons to establish a memorial to the late Lord Lister. Sir Archibald Geikie, P.R.S., was in the chair, and an executive committee was set up to recommend to a future meeting of the general committee a scheme for the memorial and to organise subscriptions. At a subsequent meeting of the executive committee arrangements were made for a public meeting to be held at the Mansion House in October, when the details of the scheme will be announced. Sir J. R. Bradford has been appointed secretary of the Lister Memorial Committee and Lord Rothschild and Sir Edward Fry are the treasurers. The executive committee includes Lords Iveagh, Rayleigh, and Rothschild, the Lord Mayor, the Lord Provosts of Edinburgh and Glasgow, Sir T. Barlow, Sir W. W. Cheyne, Sir R. J. Godlee, Sir H. Morris, Sir A. Geikie, Sir D. MacAlister, Sir W. Turner, Sir J. Wolfe-Barry, Sir A. P. Gould, Sir A. Kempe, the Hon. W. F. D. Smith, Mr. F. M. Fry, and Mr. Edmund Owen. Proposals for an international memorial are being entertained, and we trust that in considering the form which the memorial shall take certain obvious and popular subjects for endowment will be ruled out incontinently. As to research, clinical rather than experimental, and for the study of mental disease, for instance, rather than for cancer or tuberculosis should receive attentive consideration. The executive committee has a great opportunity, which, like all great opportunities, is threatened more seriously by timid counsels than by anything else. The hospital world, which, we are so glad to think, has saved the Nightingale Memorial from financial disaster, must see to it that the chance for helping on an at present neglected side of medicine or surgery is not thrown away. If hospital opinion be vigilant enough the committee of the Lister Memorial will be vigilant too.

### STAFF QUALIFICATIONS AT MELBOURNE.

To strengthen the Alfred Hospital, Melbourne, as a teaching school for medical students, the managers recently adopted resolutions that those appointed to the honorary medical and surgical staff shall have the highest degrees; that in making appointments of indoor and outdoor physicians and surgeons preference be given to specialists; and that where general practitioners are appointed to the indoor staff such appointments be conditional on their becoming specialists in their particular branch within twelve months of their appointment. The result has been that the Council of the Victorian Branch of the British Medical Association has protested its strong disapproval "of the resolutions passed by the board, and consider that they are impracticable, unworkable, and not followed by other similar institutions in Melbourne, and may inflict an injustice on medical men doing general practice. Further, they are of opinion that such resolutions are not in the best interests of the hospital."

### A SPECIAL SERIES OF RADIOGRAPHS.

In the next issue of The Hospital a Special Supplement on art paper will be included. The Supplement will comprise six radiographs which were made by Dr. A. C. Norman, of the Sunderland Eye Infirmary, in 1909 with a 12-inch coil. These radiographs are specially interesting, partly because of the skill with which they were originally made and of their subject-matter, and also because no pains or expense have been spared in their reproduction. The price of The Hospital, including the Special Supplement, will be, as usual, one penny.

### THE INADVERTENCE OF A HOSPITAL SOLICITOR.

St. Austell Cottage Hospital is faced with the loss of a legacy of £500 or else with the necessity of carrying the matter to the High Court. The late Mrs. Dodge left £500 to the local hospital provided always that a scheme were presented to the trustees, Mr. John Stephens and Mr. W. R. Kneebone, and, further, that a formal application were made for the money within a specified time. Everyone, however, including Mr. Stephens, a solicitor and first chairman of the hospital's committee, failed to realise the importance of this clause, with the result that the legacy has lapsed automatically, and, unless it can be averted, will pass to the vicar and churchwardens for church purposes. These, the residuary legatees, are understood not to press their claim, and as the loss of the legacy would place the hospital in an unfortunate position, it is hoped that if matters have to go before the High Court it will be rather a friendly arbitration than a struggle between two opposed parties. While sympathising with Mr. Stephens and his house committee in the inadvertence which is the cause of the trouble, it must be repeated once more how careful hospital officers must be in fulfilling the formalities which devolve upon trustees under wills. The fact of a solicitor having been chargeable with inadvertence shows that even solicitors are subject to the frailties of other men. The friendly attitude of the churchwardens is perhaps attributable to the fact that the testator was the daughter of a former vicar of St. Austell.

### A SANATORIUM'S PETITION.

The authorities of King Edward VII. Sanatorium have drawn up a petition praying for the grant of a charter of incorporation, which has been presented to his Majesty in Council, and which has been referred to a committee of the Lords of the Council. Formal notice, therefore, has been made that all petitions for or against such a grant should be sent to the Privy Council on or before August 19 next. As our readers are aware, a grant of incorporation confers certain legal privileges; the grantee can sue or be sued, and its possession of a seal bears witness to its new responsibilities as a legal entity.

### THE SALARIES OF INVESTIGATORS.

Perhaps the most notable feature of the Executive Committee's report of the Imperial Cancer Research Fund is their recommendation to pay higher salaries to scientific investigators. This recommendation, which has been adopted, it is hoped will be sufficient to free the best holders of such posts from any desire to seek other appointments and will attract original workers of the first order. The new laboratories provided by the Royal Colleges of Physicians and Surgeons are reported to be entirely satisfactory, and the expiring lease of the farm where the larger animals are kept is to be renewed. Sir Henry Morris has resigned the post of honorary treasurer, and Sir William Watson Cheyne is his successor. Dr. Bashford, the superintendent, has been studying Professor Wassermann's experiments in Germany, and has been lecturing by invitation on cancer in Amsterdam, Leeds, and London. In October he will deliver the Middleton-Goldsmith Lectures in New York. Dr. W. H. Woglom has been appointed first assistant of the Crocker Fund in New York. The year's investigations appear to show that in a proportion of cases inoculated cancer perishes after attaining a certain growth, and they have been directed chiefly to discover what conditions enable animals to resist the inoculation of cancer. To discover what renders the malignant cell susceptible to inimical influences rather than to induce these is suggested as the proper study to be pursued. To avoid neglecting clinical and pathological studies in the human subject cancer of the tongue has been chosen for special research. Sir William Church hopes that the experiments on mice will enable conclusions to be drawn at the end of the year as to how far hereditary predisposition to the disease exists. Professor G. Sims Woodhead was re-elected a member of the Executive Committee and Dr. William Bullock was elected a member.

### MOUNT VERNON HOSPITAL AND THE TUBERCULIN LEAGUE.

At the second annual meeting of the Tuberculin Dispensary League Dr. Camac Wilkinson's report came up for discussion. He stated in it that his method was now being practised at Brompton, Mount Vernon, Victoria Park, and Margaret Street Hospitals for Consumption. The League did not clamour for the disestablishment and disendowment of existing sanatoria. They merely urged, he continued, that the system should be tested and judged before more money was allocated to institutions which could never seriously grapple with the treatment of tuberculosis among the masses. At a cost of £350, said the report, they had successfully treated at the dispensary more cases than Mount Vernon Hospital could do in the same time at a cost of many thousands, and Mr. Lloyd George, by means of first-class hotels, might give sanatoria benefits to one out of twenty or thirty domestic servants suffering from tuberculosis. If the Insurance Commissioners would give the League the opportunity, they could successfully treat at the dispensaries fifteen out of every twenty domestic servants suffering from the same disease, and at far less cost.—These statements are sufficiently definite to provoke discussion, and it is instructive to have the opinions of the Mount Vernon Hospital upon them. Mr. W. J. Morton, Secretary of the Mount Vernon Hospital for Consumption, at once wrote: "The remarks of Dr. Camac Wilkinson in his attempt to compare the cost of the work of the League with that of this hospital [are] valueless. In the first place, both in- and out-patients are received and medically treated here. They are not necessarily consumptives, however, for sufferers from all kinds of chest disease are eligible and are admitted. Any and every form of treatment is practised which is available and which is likely to improve the health of the individual. Indeed the work is so different in character that any attempt at comparison is out of the question."

### STREET COLLECTIONS IN PARIS.

A Paris correspondent sends us the following description of the recent French street collection: "On the National fête days the Société de Secours aux Blessés Militaires held a general collection, which was energetically made by six thousand young ladies in thirty-two districts of the city for the wounded soldiers in Morocco. Instead of a wild rose, however, the badge was a tricolour flag, surcharged with a Geneva cross and the word ' Maroc,' and fixed on a pin." More than two million such little badges were sold and attached to people's buttonholes, adding 110,000 francs to the fund, of which about nine-tenths were paid in pence.

### FORTHCOMING HEALTH DISCUSSIONS AT YORK.

On the 29th inst. the members of the Royal Sanitary Institute hold at York their annual Congress. This is the twenty-seventh successive year they have foregathered in session, and the meetings have always been among the important events of the year so far as hygienic and social interests are concerned. As to the Conference, we have said already a word in advance; the principal papers deal with subjects that at the present moment should appeal to the general public as well as to those who are professionally interested in their discussion. Professor Karl Pearson will give the annual lecture, taking "Eugenics and Public Health" as his theme, and we may expect to hear something definite from him in connection with the intricacies of the problem of national betterment. Professor Kenwood will deliver the popular lecture on "The Healthy Home." The crusade against consumption supplies the subject for one discussion which will centre round dispensaries and tuberculosis treatment. In another section the medical inspection of children under school age will be dealt with; in a third the large class of industrial diseases and their prevention. Conferences have been arranged at which informal discussions will be held on other vital questions, such as housing and town planning and tuberculosis among cattle. Altogether there is a long agenda paper, which ought to give scope to all the members to display their practical interest in the sanitary and health questions of the day. The Congress closes on August 3.

### THE KENSINGTON BABY CLINIC.

In the autumn of 1911 a Baby Clinic was established at 12 Telford Road, North Kensington, in a district which was eminently in need of such an institution. The house stands in a very poor district, and the rooms have been furnished and equipped entirely by private supporters, who have befriended the Clinic in memory of the social work carried on by the late Mrs. MacDonald and Mrs. Mary Middleton. During the past year the Clinic has done really excellent work. Dr. Ethel Bentham and Dr. Kann are the consulting physicians, who attend on Mondays and Thursdays. In the first three months they have had 250 children under treatment, and the average daily attendance has now risen to 36 or more. The ailments are nearly all the diseases of malnutrition. "It is early to speak of results, but in all these things the daily care by nurse and the food-medicines, such as cod-liver oil and malt, make our recoveries much quicker than weekly attendance at hospitals gives. As time goes on we hope that prevention will largely take the place even of cures, but that lies in the future. At present there is plenty of work in mending, the number of patients is still increasing, and we must look forward to another doctors' day, and most of all, provision for a dentist to take an important work which we can scarcely touch." This is the great need mentioned in the report, and it is an urgent need. The Clinic wants regular subscribers, visitors who will interest themselves in its work, and friends who will support its endeavours. We can cordially vouch for the good work that it has done during the past year in a field that has been practically untouched. The hon. treasurer is Mrs. P. Nodin, of "Minook," Kenley, Surrey. To keep the Clinic going, a sum of £400 per year is needed, and at present the committee is only assured of £40.

### THE REMOVAL OF KING'S COLLEGE HOSPITAL.

The gift of £50,000 promised recently by an anonymous donor towards the removal of King's College Hospital to South London has just been completed, and the balance due—namely, £20,000—has been received by the committee. The sum of £4,000 is to be allotted by request to the building of the medical school. The committee for the removal of the hospital is to be congratulated on the completion of this gift, which has been handed over in full with so little delay after the donor's intentions were announced.

### HECKLING TO NO PURPOSE.

A recent meeting of the Conway and Penmaenmawr Joint Hospital Board led to the passing of a resolution disapproving of the conduct of the board's engineer, Mr. T. B. Farrington, of Llandudno. Mr. J. W. Raynes, with reference to the board's newly opened hospital, questioned Mr. Farrington as to the efficiency of the electric plant and of an oil engine which is used to charge the accumulators. A desultory discussion then arose as to the contractor's claim for an amount in excess of that allowed by Mr. Farrington. In the course of it Mr. Farrington said that he must leave shortly to catch a train, as he had been three hours at a previous meeting, and for a family reason it was important to him to leave for Llandudno. Mr. Raynes thereupon remarked that "Mr. Farrington should remain at our pleasure," and in answer to the engineer's statement that he did not wish to waste his time or the board's, Mr. Raynes retorted, "Our time is more valuable than yours, which you get handsomely paid for." Whereupon Mr. Farrington said "Good afternoon," and left the room. Mr. J. P. Griffith supported Mr. Raynes in condemning this departure as disrespectful to the board, whereupon the Chairman, Mr. P. H. M'Clement, had remarked that there had been a good deal of heckling to no purpose. After further discussion a motion disapproving of Mr. Farrington's action in leaving was carried. To the Chairman's comment we believe our readers will agree there is nothing to add.

### A GARDEN CITY HOSPITAL.

Letchworth, one of the first garden cities to be established, has recently decided to provide itself with a cottage hospital. Mr. D. B. Cockerell was in the chair at the meeting which arrived at this decision, and announced that if the project were favoured Sir Henry Burdett had written to say that if the locality desired him to do so, he would give an address explaining its needs. Mr. Cockerell remarked that the whole Garden City project was a health scheme, but, as sickness inevitably remained, a nursing association, a health visitor, and a dental scheme had already been undertaken. Mrs. Craske, who has worked very hard, has undertaken to pay the cost of building and maintaining the hospital. In 1906 the committee of the provident dispensary included a cottage hospital in its objects. This committee already has purchased an ambulance. Next year will be the tenth birthday of the Garden City, when the erection and opening of a cottage hospital is hoped to be the chief celebration of that event. The scheme includes an institution of eight public and four private beds, which will also be the centre of treatment of a school clinic. The cost is expected to be £2,000, and £3,500 is required as a partial endowment fund. We entirely agree with Mrs. Craske's wise warning that an endowed cottage hospital is not altogether a good thing, and in case any members of the Garden City are not convinced of this we refer them to the paragraph entitled "A Cottage Hospital not to be Envied," which appeared in this column on July 13.

### TALL HATS AND TRADE UNIONISM.

Not the least interesting feature of the opening day's debate at the representative meeting of the British Medical Association at Liverpool last week was the closing discussion on the desirability of converting the Association into a body registered as a trade union. Recent events, of course, have brought this question prominently to the front, and perhaps the chief difficulty in the way of clear thinking on the subject is the obsolescent superstition that it is derogatory to professional men to have a recognised trade union. "Recognised," we say, for, of course, in practice, no matter what its status may be, a large body of men engaged in the same profession and with corporate interests has found in practice that it is essential to have a corporation to control them. The Law Society and the Society of Authors are very strong trade unions, and seem to show how well the principles of trade unionism have been honoured in practice by the professional classes. Whether this be called a trade union or a professional organisation is a matter of names, except that the shorter and more historic title is more free from objection, and can be recognised more simply. Besides, tall hats and trade unionism seem to understand their relations better than trade unionism and corduroys, for to an attentive observer nothing is more startling than the contrast between the doctors' control of their representatives and the apparently unchecked domination of the rank-and-file workmen by theirs. The latter, indeed, have assumed the names of leaders: a name which, judging by a stormy meeting at the Queen's Hall some months ago, the doctors would never allow their delegates to usurp. However, such general considerations apart, the upshot of the present discussion was a postponement for a year, during which, it may be hoped, that clear thinking will prevail.

### THE PUBLIC RESPONSIBILITIES OF MEDICINE.

Professor Benjamin Moore, who has already done so much to stir up the medical profession to a realisation of its public responsibilities, is the leader in a movement which has for its main object the foundation of a State Medical Service on really efficient lines. Professor Moore has received cordial support from many prominent members of the profession and encouragement from every section. His present intention is merely to establish a State Medical Service Association with local branches in every district. The main object of the Association will be "to promulgate the advisability of a State Service among the medical profession and to make clear its advantages to the public and to promote its adoption by Parliament." The basis of its programme is outlined as follows: "The whole profession shall be organised on the lines of other State Services now in existence; entry to the profession shall be by one State examination only; each member of the Service shall be paid an adequate salary, increasing gradually, according to length of service and position, and shall be entitled to a pension after a specified number of years' work, or in the case of permanent disablement; whilst members of the public shall have free choice of doctors, no doctor shall be called upon to have charge of more than a specified number of patients; one of the primary objects of the State Service shall be to unite preventive and curative medicine." With regard to the other suggestions—hospital nationalisation for instance—there will be a wide divergence of opinion. But in any case Professor Moore's Association should stimulate public attention in national health questions, and we wish it every success.

### THIS WEEK'S DRUG MARKET.

Changes in the value of drugs have been uncommonly few during the week, and business continues very quiet. It is thought probable that a reduction in the price of opium will take place shortly; in the meantime the demand is very slow as buyers are waiting for the expected reduction. Morphine is also tending lower in value. The price of buchu leaves is well maintained. Carbolic acid tends rather lower in value, and, as a consequence, zinc sulphocarbonate and sodium sulphocarbonate have been slightly reduced in price. It appears probable that the price of thymol will be advanced. Citric and tartaric acids tend dearer. Oil of cloves and oil of carraway are quoted higher. The position of cod-liver oil is unchanged. There is a good inquiry for eucalyptus oil, and the price is well maintained, with the probability of an advance. Ipecacuanha is slightly dearer. Essence of lemon is advancing in price. Very high prices are asked for glucose.

